# Phase-Controlled Synthesis and Phase-Change Properties of Colloidal Cu–Ge–Te Nanoparticles

**DOI:** 10.1021/acs.chemmater.4c01009

**Published:** 2024-06-24

**Authors:** Dhananjeya Kumaar, Matthias Can, Helena Weigand, Olesya Yarema, Simon Wintersteller, Rachel Grange, Vanessa Wood, Maksym Yarema

**Affiliations:** † Chemistry and Materials Design, Institute for Electronics, Department of Information Technology and Electrical Engineering, ETH Zürich, 8092 Zürich, Switzerland; ‡ Optical Nanomaterial Group, Institute for Quantum Electronics, Department of Physics, ETH Zürich, 8093 Zürich, Switzerland; § Materials and Device Engineering, Institute for Electronics, Department of Information Technology and Electrical Engineering, ETH Zürich, 8092 Zürich, Switzerland

## Abstract

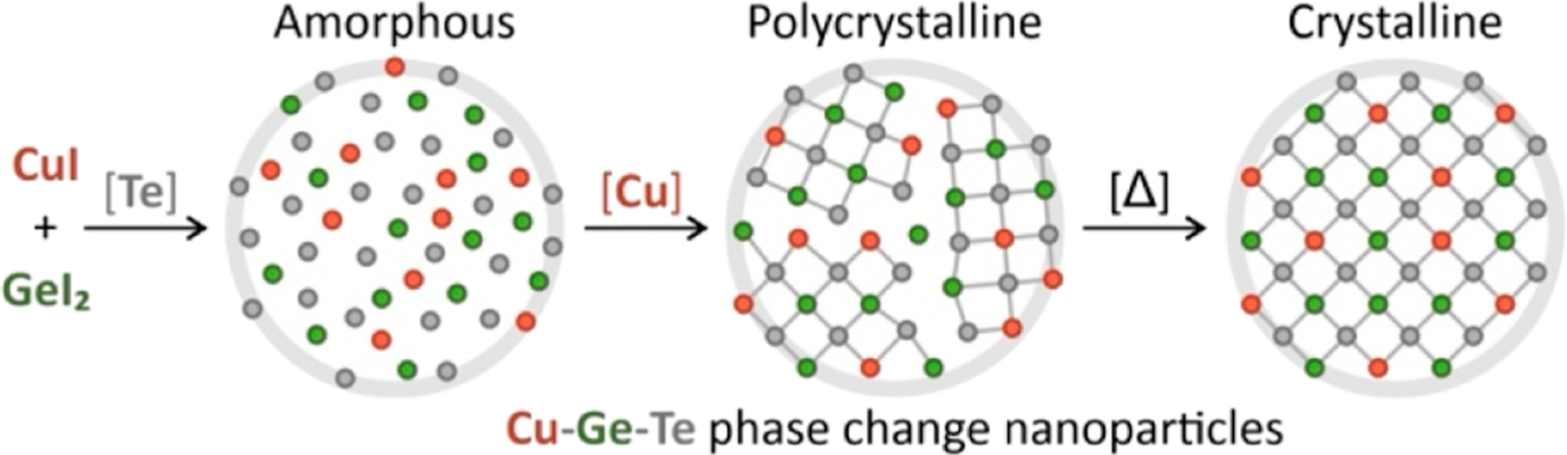

Phase-change memory (PCM) technology has recently attracted a vivid interest for neuromorphic applications, in-memory computing, and photonic integration due to the tunable refractive index and electrical conductivity between the amorphous and crystalline material states. Despite this, it is increasingly challenging to scale down the device dimensions of conventionally sputtered PCM memory arrays, restricting the implementation of PCM technology in mass applications such as consumer electronics. Here, we report the synthesis and structural study of sub-10 nm Cu–Ge–Te (CGT) nanoparticles as suitable candidates for low-cost and ultrasmall PCM devices. We show that our synthesis approach can accurately control the structure of the CGT colloids, such as composition-tuned CGT amorphous nanoparticles as well as crystalline CGT nanoparticles with trigonal α-GeTe and tetragonal Cu_2_GeTe_3_ phases. In situ characterization techniques such as high-temperature X-ray diffraction and X-ray absorption spectroscopy reveal that Cu doping in GeTe improves the thermal properties and amorphous phase stability of the nanoparticles, in addition to nanoscale effects, which enhance the nonvolatility characteristics of CGT nanoparticles even further. Moreover, we demonstrate the thin-film fabrication of CGT nanoparticles and characterize their optical properties with spectroscopic ellipsometry measurements. We reveal that CGT nanoparticle thin films exhibit a negative reflectivity change and have good reflectivity contrast in the near-IR spectrum. Our work promotes the possibility to use PCM in nanoparticle form for applications such as electro-optical switching devices, metalenses, reflectivity displays, and phase-change IR devices.

## Introduction

In consumer electronic devices, such as smartphones, more than 60% of energy is spent on data movement between the processing and memory units due to von Neumann architecture in computing systems, which limits the bandwidth and rate of accessing the data.^1,2^ Phase-change memory (PCM) may revolutionize the way we compute, being a promising candidate for in-memory computing, i.e., when the data storage and logical operations take place within a single block.^3^ PCM has already seen great success as a storage class material in optical discs due to the stark contrast in reflectivity between the amorphous and crystalline phases.^4^ In addition, their ability to show a strong contrast in electrical resistance between the two phases has paved their way into electronic memory devices, enabling it as a viable emerging memory technology which is CMOS-compatible, two-terminal, and multilayered.^5^ For optical applications, a laser pulse is used to read and write data, whereas in electronic applications, an electrical pulse is applied to induce strong Joule heating, enabling a phase change in the material. Recent developments in PCM devices and materials engineering have widened the application range to neuromorphic hardware,^6,7^ electro-optical modulators,^8−11^ and even space applications.^12^

Currently, the most widely used PCM layer is a sputtered Ge–Sb–Te alloy (such as Ge_2_Sb_2_Te_5_ or GST225) offering a balanced optimum of crystallization speed, cycling stability, intermediate states, and relatively low power consumption.^13,14^ However, from a material standpoint, GST225 has suboptimal thermal stability due to a low crystallization temperature of 170 °C and is therefore unsuitable for high operating temperature applications. Furthermore, GST alloys are characteristic of relatively large, >6% density difference between the amorphous and crystalline phases,^15^ limiting the endurance and switching speed of GST devices. In this regard, doping strategies have been used for telluride PCM materials in order to increase the crystallization temperature and to improve the resistance drift, mechanical stability, resistance contrast, and switching speed.^16−19^ For example, Cu–Ge–Te (CGT) PCM materials have been shown to require a lower switching energy while providing higher thermal stability than GST at comparable switching speeds.^20^ Furthermore, the density change of CGT upon crystallization is smaller with respect to GST or GeTe, which is beneficial for the cycling properties of PCM devices.^21^ Finally, the CGT material features a very peculiar characteristic: its crystalline phase has a lower optical reflectivity than the amorphous phase, an opposite trend of typical PCM materials.^22^ The negative reflectivity contrast of CGT can be leveraged for tandem memory cells in combination with traditional PCM materials for unconventional logic. Nevertheless, there is a limited understanding of the role of d-block dopants, such as Cu, in improving the switching speed, thermal stability, and reflectivity contrast.

One of the reasons why materials discovery is largely restricted for PCM technology is fabrication limitations. PCM layers are commonly deposited as thin films by sputter deposition or physical/chemical vapor deposition, which are intrinsically highly specialized, material-specific, and expensive process techniques.^23^ In this regard, solution-based processing has earned a reputation as a relatively straightforward and inexpensive approach for materials screening, offering a feasible and fast alternative to create new PCM compositions.^24−28^ Specifically, colloidal hot-injection method has been proven to be a rapid and convenient way to produce nanoscale chalcogenide PCM materials, such as GeTe.^29−32^ For example, excellent size control has been achieved for binary GeTe nanoparticles,^31^ offering the advantage of size-dependent tunability of crystallization temperature.^33^ Synthesizing ternary and multicomponent PCM nanoparticles, however, comes with the challenge of simultaneous size and composition control,^34^ which is exacerbated when combining aliovalent elements.^35,36^ The use of elemental precursors with different reactivities is often required to engineer ternary nanoparticles, limiting the extent and precision of composition control. This makes it cumbersome to build a broad synthesis framework that enables materials screening of multicomponent chalcogenide nanoparticles. Recently, we resolved this challenge, developing a generalizable amide-promoted colloidal synthesis approach for telluride nanoparticles.^37^ Our method enabled a family of new PCM colloids, such as Sn–Ge–Te, Bi–Ge–Te, Pb–Ge–Te, or In–Ge–Te, with excellent composition control. However, the synthesized phase of nanoparticles, whether amorphous or crystalline, is predefined by the crystallization properties of bulk materials. Therefore, in order to achieve amorphous Sn–Ge–Te, we needed to carry out a two-step synthesis to include amorphous GeTe nanoparticles as an intermediate, followed by cation-exchange reaction with the reactive Sn precursor.^37^

In this work, we take the CGT system as an example and present a simple yet effective hot-injection synthesis for colloidal nanoparticles with full chemistry control of the product. In particular, we study the kinetic parameters that are necessary to achieve phase and composition tuning in the CGT nanoparticles as well as narrow size distributions and a small sub-10 nm size range of the product. We also observe the phase conversion from an amorphous to crystalline CGT structure upon synthesis. Eventually, we map the chemical and kinetic parameters required to produce CGT nanoparticles in amorphous, trigonal α-GeTe, and tetragonal Cu_2_GeTe_3_ phases. We then study the structure and crystallization mechanism of CGT nanoparticles using X-ray diffraction (XRD) and X-ray absorption spectroscopy (XAS). Finally, we perform ellipsometry characterization of CGT nanoparticle thin films capped with inorganic ligands, confirming the reflectivity contrast between the amorphous and crystalline states, which is particularly promising in the near-infrared spectrum. These results can be translated to applications such as quantum dots integrated onto waveguides for telecommunication applications, metalenses, and phase-tunable photodetectors.

## Experimental Section

### Synthesis of Cu–Ge–Te Nanoparticles

In a typical small-scale synthesis, GeI_2_ (0.039–0.354 mmol) was dissolved in 7.5 mL of TOP, and CuI (0.033–0.295 mmol) was dissolved in 3 mL of TOP in the glovebox. The two solutions were then transferred to a dried three-neck flask under vacuum, connected to the Schlenk line. The mixture was dried at 110 °C for 30 min. The flask was filled with nitrogen before injecting the mixture of TOP:Te (0.8 mL of 1 M stock solution) and LiN(SiMe_3_)_2_ (0.5 mL of 1.6 M stock solution). To obtain phase-specific CGT nanoparticles, both the temperature and reaction time were precisely controlled. The composition of CGT nanoparticles was proportional to the initial amounts of metal salts. To obtain amorphous-phase CGT nanoparticles, the heating mantle attached to the reaction flask was maintained at 260 °C, and the reaction time was typically less than 15 min. To obtain crystalline CGT nanoparticles, longer reaction time (>15 min) and reaction temperatures up to 280 °C were required.

### Washing Procedure for Cu–Ge–Te Nanoparticles

Approximately 1 mL of oleic acid was added to the nanoparticle crude solution. To this were added 25 mL of anhydrous ethanol and 10 mL of anhydrous methanol, and the mixture was centrifuged at 8000 rpm for 5 min to precipitate the Cu–Ge–Te material. After several washing cycles, the nanoparticles were stored in anhydrous chloroform.

### Electron Microscopy

Transmission electron microscopy (TEM) and scanning transmission electron microscopy (STEM) were performed on FEI Talos 200X. For HR-TEM measurements, the nanoparticles were drop-cast onto ultrathin carbon TEM grids (Ted Pella) and annealed at 100 °C to remove the solvent and organics to lower the electron beam contamination. The elemental quantification was carried out with energy-dispersive X-ray (EDX) spectroscopy measured on an FEI Quanta 200 system.

### X-ray Methods

Powder XRD and high-temperature XRD were measured using a Rigaku Smartlab 9 kW system supplied with a Cu anode and a HyPix-3000 SL detector. For in situ high-temperature XRD, the particles were mixed with anhydrous boron nitride powder and sealed in a 1.5 mm quartz capillary tube (from Hilgenberg) in a glovebox to avoid possible oxidation. The heating rates were set to 5 °C/min, and 2θ was scanned in a small range to monitor the (202) Bragg reflection of the α-GeTe phase. XAS was measured at the SuperXAS beamline (X10DA) at the Paul Scherrer Institute. The sample preparation of the nanoparticles in boron nitride sealed in a quartz capillary is similar to in situ high-temperature XRD measurements. The capillary carrying the amorphous nanoparticles was measured at the Ge and Te K-edges at room temperature. Subsequently, the same sample was heated in a custom-made heating element beyond the crystallization temperature of the particles at 350 °C and maintained for 30 min to ensure complete crystallization. After cooling down to room temperature, the crystallized nanoparticle sample was measured once again at different energy edges to get a comprehensive understanding of the structural difference between amorphous and crystalline CGT nanoparticles.

### Optical Characterization

Absorption spectroscopy of the CGT nanoparticles was measured in liquid form, with the particles dispersed in chloroform. The solvent background was accordingly subtracted prior to fitting the Tauc equation to obtain the band gap. Fourier-transform infrared spectroscopy of the ligand-exchanged nanoparticles was measured with a ZnSe window on a Bruker V70 system equipped with an InGaAs detector. Ellipsometry of the CGT nanoparticles was measured in a thin film form post-ligand exchange to ensure better packing of nanoparticles into films. The organic ligands were substituted for an iodide shell, which ensures good stability while spin coating the nanoparticles in a polar solvent. Thin films were capped with sputtered SiO_2_ to prevent oxidation, and the ellipsometry was measured using Woollam VASE with 1 × 2 mm spot size where no visible defects were present on the sample. The data were collected between 65 and 75° at three different angles.

## Results and Discussion

### Phase Control for Cu–Ge–Te Nanoparticles

To date, only Wang et al. report colloidally stable CGT nanoparticles with Cu_2_GeTe_3_ composition, exhibiting a zinc-blende-related cubic phase and a narrow size distribution of 14.7 ± 1.0 nm.^38^ While this is an encouraging pathfinder report for us, we aim to improve upon it and develop a full synthetic platform, which allows for an accurate composition control and ultrasmall sub-10 nm sizes—a size range, where PCM properties become a function of physical nanoscale dimensions (size or thickness). Ideally, we can also control the phase of Cu–Ge–Te nanoparticles and be able to prepare either amorphous or crystalline products depending on the reaction conditions. This will enable us to study and apply the phase-change properties of CGT colloids by tuning their size, composition, and phase.

**Figure 1:**
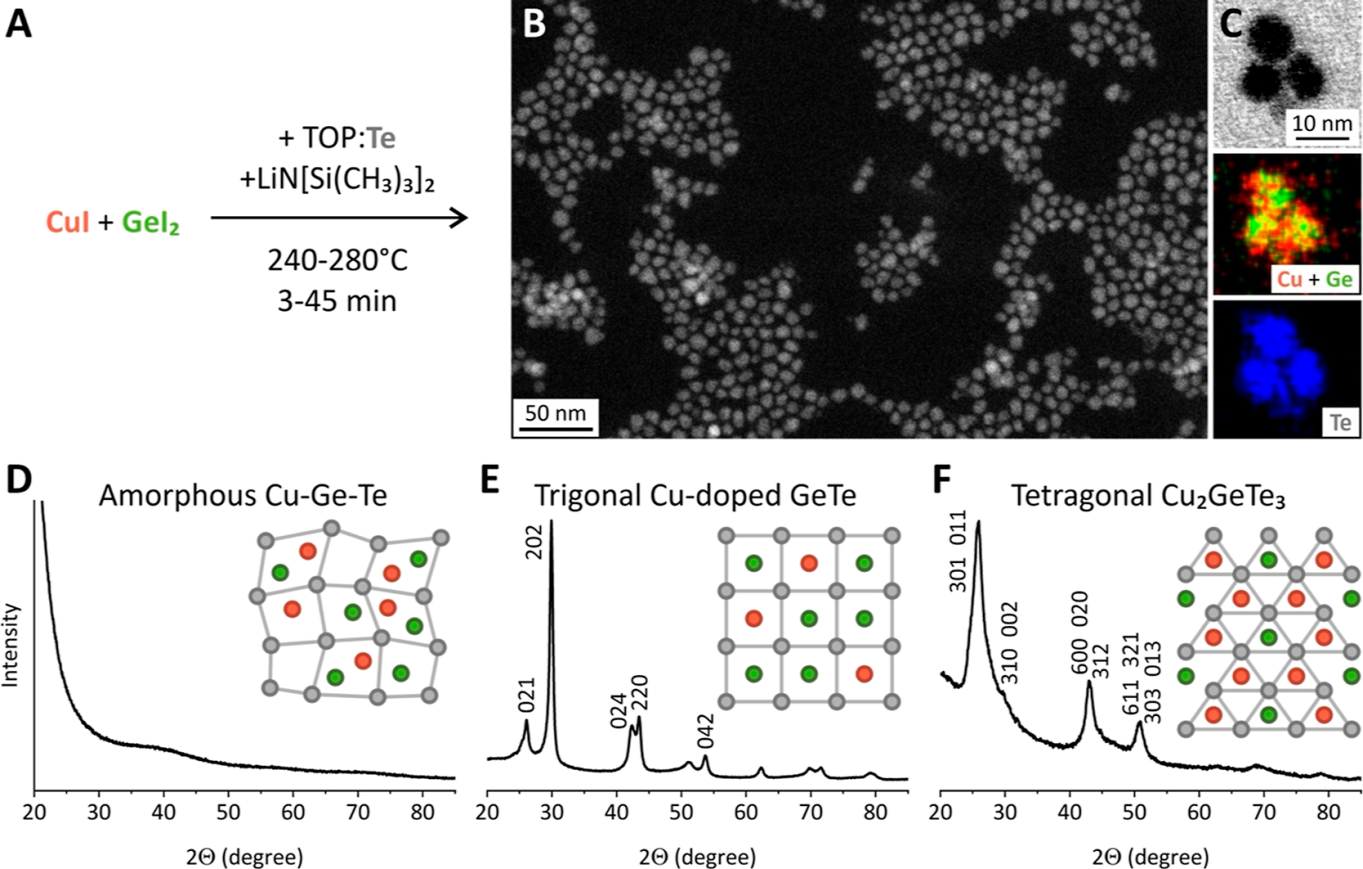
Synthesis and different phases of Cu–Ge–Te nanoparticles. (A) Reaction scheme of amide-promoted synthesis. (B) Scanning TEM image and (C) STEM–EDX composition map of CGT nanoparticles. (D–F) X-ray diffractograms of CGT nanoparticles with different phases of the product. The absence of any Bragg reflections in (D) indicates the amorphous CGT structure, while XRD patterns in (E,F) point to trigonal α-GeTe and tetragonal Cu_2_GeTe_3_ phases, respectively. Insets (D–F) illustrate the structural motifs of the Cu–Ge–Te phases.

To prepare CGT nanocrystals, we employ an amide-promoted synthesis,^39^ which is schematically illustrated in Figure 1A. In this hot-injection method, LiN(SiMe_3_)_2_ is coinjected with a chalcogen solution into the flask, containing metal precursors. The use of LiN(SiMe_3_)_2_ is crucial in our reaction because it acts as a promoter agent, converting metal precursors into reactive intermediates. This helps to improve the nucleation rate of constituent metals and hence obtain sub-10 nm CGT nanoparticles with excellent composition control. Based on the empirical outcome of our previous work,^37^ we choose germanium(II) iodide (GeI_2_) and copper(I) iodide (CuI) as the metal precursors and TOP:Te solution as a chalcogen source. We then optimize the kinetic parameters of the reaction within 240–280 °C and 3–45 min, in order to maintain a narrow size distribution (Figure 1B). EDX maps (Figure 1C) demonstrate the presence of all three constituent elements (Cu, Ge, and Te) in the composition of nanoparticles, as well as composition homogeneity within each Cu–Ge–Te nanoparticle.

Importantly, our work for the first time provides a means to prepare Cu–Ge–Te with different crystal structures. Figure 1D–F shows the representative X-ray diffractograms of amorphous, trigonal, and tetragonal Cu–Ge–Te nanoparticles. All three structures are highly relevant for PCM technology.^40−43^ For example, the trigonal CGT material (α-GeTe phase) has improved data retention properties and smaller volumetric change upon phase transitions.^20,44^ The amorphous CGT material is characteristic of an abundance of unusual threefold ring local coordination.^45,46^ Finally, the Cu_2_GeTe_3_ PCM material has a set of unconventional properties, such as negative reflectivity change upon crystallization and sp^3^-type bond hybridization.^22,47^ Nevertheless, this tetrahedrally coordinated PCM material shows fast switching characteristics also in addition to the higher crystallization temperature with respect to octahedrally coordinated GST fragile glasses. Taken together, the unconventional switching mechanism in CGT materials points to the important role of Cu 3d orbitals, enabling p–d bond mixing and delocalization of d-electrons upon phase transitions.^21,46,47^ In the next section, we discuss in detail how to achieve each of the three Cu–Ge–Te phases. We also report an experiment during which we observe amorphous-to-crystalline CGT conversion.

### Synthesis of Cu–Ge–Te Nanoparticles

**Figure 2:**
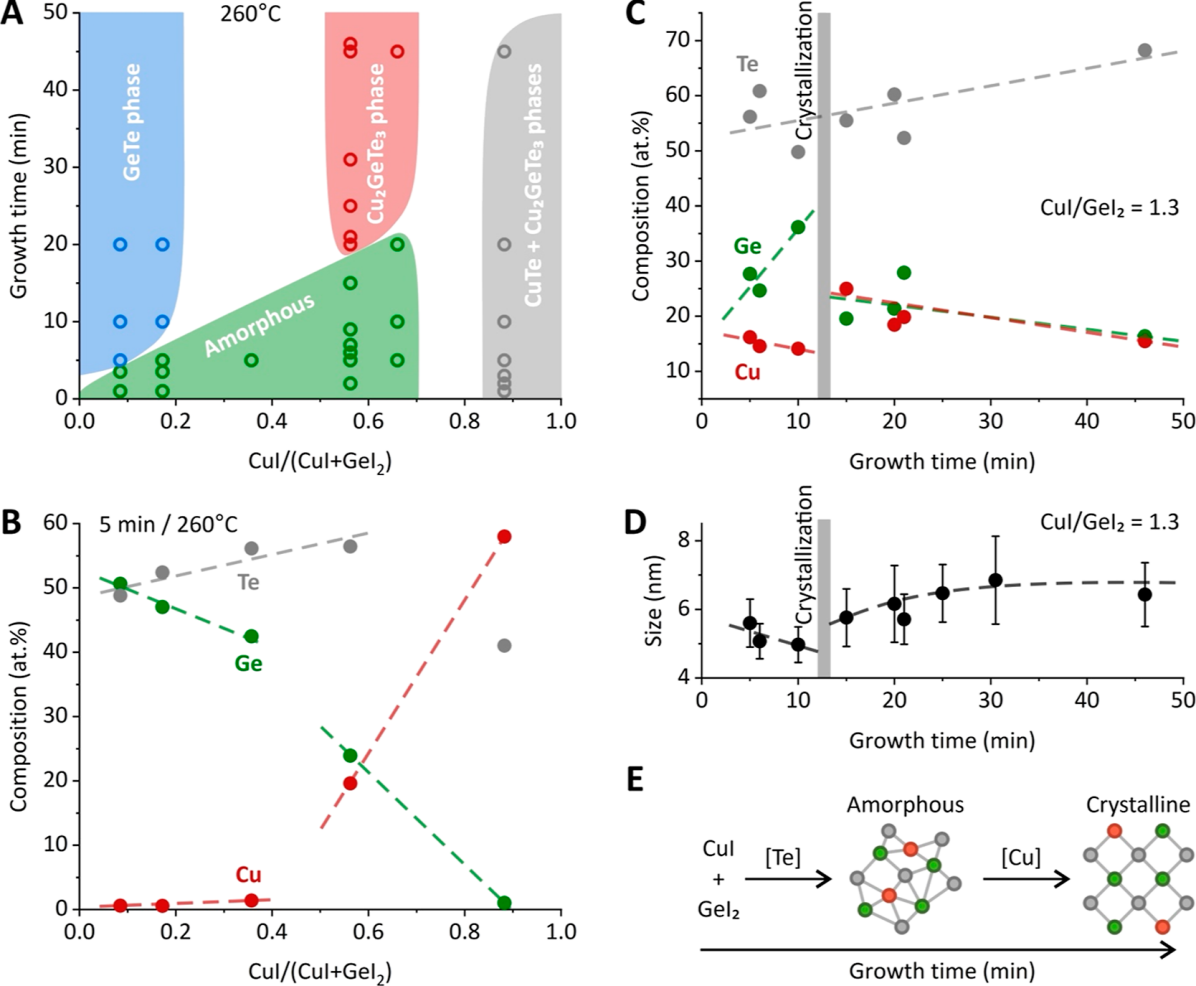
Synthesis control for Cu–Ge–Te nanoparticles. (A) Phase map for the reaction at 260 °C, illustrating the synthesis conditions to obtain CGT in amorphous, α-GeTe, and Cu_2_GeTe_3_ phases. (B) Precursor effects on the composition of CGT nanoparticles, prepared at 260 °C and 5 min of growth time. (C,D) Influence of growth time on the composition (C) and size (D) of the CGT nanoparticles. EDX and TEM quantifications have been used for (C,D), respectively. (E) Schematics of the reaction mechanism for CGT nanoparticles.

We begin to systematically explore the colloidal synthesis of CGT nanoparticles, starting from various ratios of metal iodide precursors. We also monitor the synthesis pace at different growth times to understand the reactivity of precursors and the reaction mechanism. For this initial round of experiments, we choose a constant reaction temperature of 260 °C (Table S1 summarizes the reaction conditions). We then measure each sample with XRD (Figure S1), leading to the construction of a phase diagram as a function of reaction parameters (Figure 2A). Shorter reaction times yield amorphous CGT nanoparticles (green area in Figure 2A). This is observed for any precursor ratio, except the highly CuI-rich conditions, where we register a mixture of CuTe and Cu_2_GeTe_3_ phases (gray area in Figure 2A). For longer reaction times, amorphous CGT transforms into crystalline phases. In agreement with the previously reported phase diagram,^20^ small molar fractions of CuI (up to 20 mol %, blue area in Figure 2A) result in the α-GeTe phase, while the higher CuI initial concentration (between 50 and 70 mol %, red area in Figure 2A) leads to the Cu_2_GeTe_3_ phase. Interestingly, the threshold time for the in situ crystallization of amorphous CGT nanoparticles is longer for higher CuI contents. This observation suggests that the incorporation of Cu improves the data retention properties of the GeTe PCM material, staying in agreement with previous literature findings for CGT thin films.^20^ Therefore, Figure 2A provides a direct guide to synthesize three different phases of GCT nanoparticles, namely, the amorphous CGT, Cu-doped α-GeTe, and Cu_2_GeTe_3_ nanoparticles.

To understand the reactivity balance between CuI and GeI_2_ precursors, we perform a composition series at fixed kinetic parameters, 5 min of growth time at 260 °C (Figure 2B). For GeI_2_-rich conditions, we observe a negligibly small Cu content. However, under equivalent amounts of iodides and CuI-rich conditions, the precursor ratio matches the composition of the CGT nanoparticles (Figure 2B). These results are unconventional as they cannot be attributed to the sluggish kinetics of CuI in the reaction mixture. To further investigate the interplay between the precursors, we track the composition and size of CGT nanoparticles as the reaction proceeds between 5 and 45 min at 260 °C (Figure 2C,D). During the first minutes of growth, the CGT nanoparticles become increasingly Cu-deficient. This trend is even more pronounced at 240 °C, where the Cu content drops to zero at 10 min of growth (Figure S2). We therefore hypothesize that the initially formed CGT nanoparticles may gradually lose Cu ions, e.g., to the TOP complex agent, as was reported earlier for Cu_2–*x*_Se nanoparticles.^48^ In line with this hypothesis is the fact that the size of CGT nanoparticles decreases proportionally to Cu deficiency. However, for 15 min of reaction time, we observe an abrupt increase of Cu content (Figures 2C and S2). Simultaneously, the size of the CGT nanoparticles increases by approximately 1 nm, on average (Figure 2D). Furthermore, this sudden influx of Cu atoms is followed by the crystallization of amorphous CGT nanoparticles (between 15 and 20 min, Figure 2A). We attribute these observations to the higher thermodynamic stability of crystalline phases. Apparently, Cu atoms are initially weakly bonded in the amorphous CGT structure, leading to the leakage of Cu ions in the reaction medium. However, after a given threshold time when CGT crystallizes, Cu ions are returned back to the structure, matching the equilibrium composition of the targeted phase (Cu_2_GeTe_3_ for the case in Figure 2C). Figure 2E illustrates our understanding of the CGT synthesis schematically via the formation of an amorphous intermediate, followed by a Cu-enabled in situ crystallization process.

**Figure 3:**
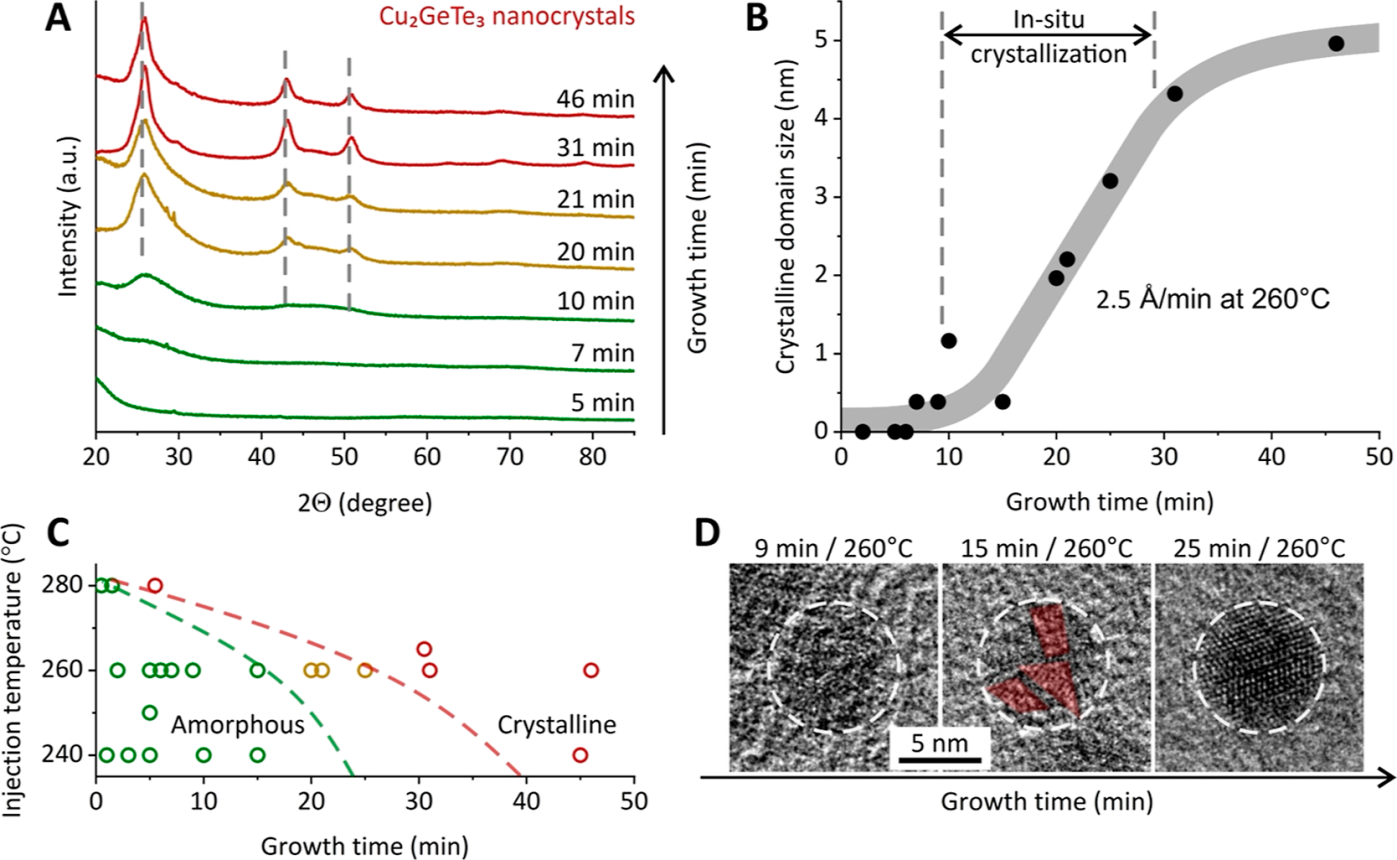
In situ crystallization of Cu–Ge–Te nanoparticles. (A) XRD patterns of CGT nanoparticles at 260 °C and different growth times and (B) extracted crystal domain size via the Scherrer analysis of the main Bragg reflection. (C) Phase diagram, mapping amorphous and crystalline states of CGT nanoparticles across injection temperature and growth time as well as (D) corresponding TEM images of the states.

We proceed to study the crystallization kinetics occurring during the growth of CGT nanoparticles. For this purpose, we collect XRD patterns at different reaction times, yet consistent reaction temperature of 260 °C (Figure 3A). As expected, CGT nanoparticles are amorphous immediately after nucleation, which is indicated by the absence of Bragg reflections for a growth time of 5 min. As the reaction continues, the broad Bragg reflections of tetragonal Cu_2_GeTe_3_ phase appear and become progressively sharper, manifesting a slow crystallization of CGT nanoparticles. We then estimate the crystal domain size, fitting a Gauss peak function to the main Bragg reflection and applying a Scherrer equation under the assumption of a nearly spherical shape of the crystal domain (i.e., the entire nanoparticle). The results, plotted in Figure 3B, suggest that the crystallization onset is around 10 min, and it takes approximately 20 min to complete the crystallization process. After 30 min of growth, the width of Bragg reflections saturates, corresponding to a crystalline domain of 5 nm, i.e., approximately the size of the CGT nanoparticles (Figure 2D). Hence, we complete our mechanistic model (Figure 2E) with the timeline of the crystallization process: After 10 min of induction time, CGT nanoparticles crystallize gradually with an average rate of 2.5 Å/min (Figure 3B).

We extend the crystallization kinetics study to different reaction temperatures. Figure 3C presents a map of the apparent state of the CGT nanoparticles. As expected, the crystallization process is slower at a lower reaction temperature of 240 °C, where we register the crystalline CGT phase only after 45 min of growth. On the contrary, the higher temperature of 280 °C results in a crystalline CGT structure already after 5.5 min of reaction time. The TEM analysis (Figure 3D) complements our XRD data, showing three snapshots of CGT products after 9, 15, and 25 min of growth at 260 °C. For 9 min of reaction time, the CGT nanoparticles exhibit an amorphous structure, which is demonstrated by the seemingly random ordering of atoms. At 15 min of growth, CGT nanoparticles are highly polycrystalline with a few visible crystalline domains of 1–2 nm (shaded red areas in Figure 3D). Finally, after 25 min of growth, CGT nanoparticles exhibit excellent crystallinity. Thus, CGT nanoparticles represent a unique system, for which the crystallization process can be conveniently monitored by high-resolution TEM methods, allowing accurate studies on the atomic scale.

### Crystallization of Cu–Ge–Te Nanoparticles

**Figure 4:**
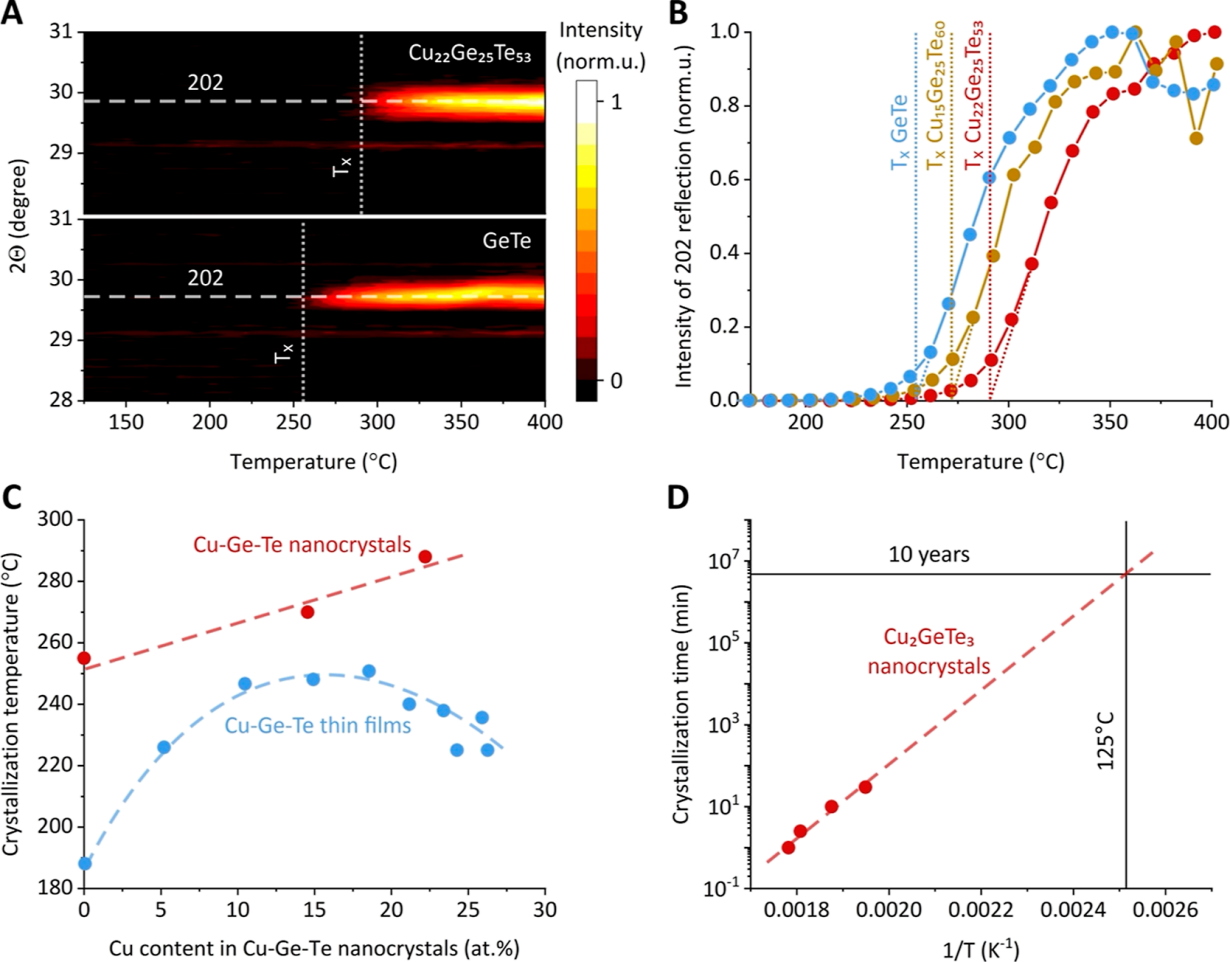
High-temperature structural dynamics of Cu–Ge–Te nanoparticles. (A) In situ heating XRD measurements and (B) intensity of (202) Bragg reflection of the α-GeTe phase for the GeTe and CGT nanoparticles, showing the composition-dependent onsets of crystallization. (C) Nanoscale effect on the crystallization temperature of CGT nanoparticles in comparison to bulk thin films. (D) Arrhenius plot, estimating a nonvolatility temperature for Cu_2_GeTe_3_ nanoparticles.

It is widely accepted that the crystallization temperature of a PCM material is composition-dependent (e.g., it ranges between 170 and 310 °C within the GST system).^49^ In addition to this, nanoscale effects are observed.^50^ Specifically, higher crystallization temperatures (with respect to the bulk thin films) have been reported for GeTe and Sn_*x*_Ge_1–*x*_Te colloidal nanoparticles.^37,51^ This has been attributed to the size-dependent crystallization entropy.^31^ More recently, the nanoscale influence on structure dynamics has been discovered: the amorphous structure of GeTe-based PCM materials appears to be more stable at the nanoscale, due to weaker β-relaxation processes and consequently reduced aging.^33^ To understand the influence of size and composition on the crystallization temperature of the CGT materials, we synthesize three samples of amorphous nanoparticles with the compositions of Cu_22_Ge_25_Te_53_, Cu_15_Ge_25_Te_60_, and GeTe, and similar sizes of 5–6 nm. The crystallization onset of nanoparticles was then measured by in situ high-temperature XRD measurements with the constant heating rate of 5 °C/min. All three samples crystallize at high temperatures to the trigonal α-GeTe phase, which is manifested by the appearance of (202) reflection at a 2θ degree of 29.7–29.8 (Figure 4A). Figure 4B plots the intensity profiles of the (202) peak against the temperature, from which we can estimate the crystallization temperatures of GeTe and CGT nanoparticles as the onsets of sigmoidal growth curves.

We also perform XAS measurements of CGT nanoparticles (with 5 at. % Cu) before and after annealing at 350 °C. Our XAS results (Figure S3 and Tables S2 and S3) support the XRD data, showing trigonal α-GeTe phase for CGT nanoparticles, with a fraction of Ge–Te bonds replaced by Cu–Te bonds. The amorphous CGT structure also contains roughly the same number of Cu–Te bonds, albeit slightly shorter than in the crystalline CGT phase. Compared to GeTe nanoparticles,^33^ the CGT structure contains a notably lower amount of Ge–Ge homopolar bonds, both in amorphous and crystalline phases. This suggests fewer defect states in the CGT material, leading to higher stability and better data retention of CGT materials, as previously proposed the in literature.^44^

Comparing CGT nanoparticles with their bulk counterpart,^20^ we can draw two important conclusions. First, the trend for the crystallization temperature of CGT nanoparticles with the Cu content is different to the one observed for bulk GCT films (Figure 4C).^20^ While adding 15 atom % of Cu increases the crystallization temperature for both bulk and nano-CGT, further increasing the Cu content leads to the opposite effect. This may be associated with the large surface area of nanocrystals that are Cu-rich. Such scenario is likely, given the observation of easy Cu-ion mobility in the reaction mixture (Figure 2D). Therefore, we hypothesize that the Cu content in the CGT core is overestimated by the EDX analysis (Figure 4C), rendering both trends (for bulk and nano) more alike. Importantly, however, the crystallization temperature of CGT nanoparticles is always higher than that of the bulk, which matches our previous observations for GeTe and Sn–Ge–Te nanoparticles.^31,37^

Finally, we can estimate the nonvolatility of CGT nanoparticles, plotting the time required to crystallize during the reaction (Figure 3C) versus inverted temperature. This dependence shows the typical Arrhenius behavior (Figure 4D). By extrapolating this dependence, we observe that the nonvolatility benchmark of 10 years’ structure stability holds true up to 125 °C for CGT nanoparticles, making our material suitable for a large range of high-temperature memory applications, from industrial chips operating at harsh environments to automotive industry and high-power applications. Our estimate of CGT nonvolatility stays in agreement with the literature, reporting a higher thermal stability of amorphous Cu_2_GeTe_3_ thin films, which is attributed to the large bond enthalpy of the structure.^40^

### Optical Phase-Change Properties of Solution-Deposited Cu–Ge–Te Thin Films

**Figure 5:**
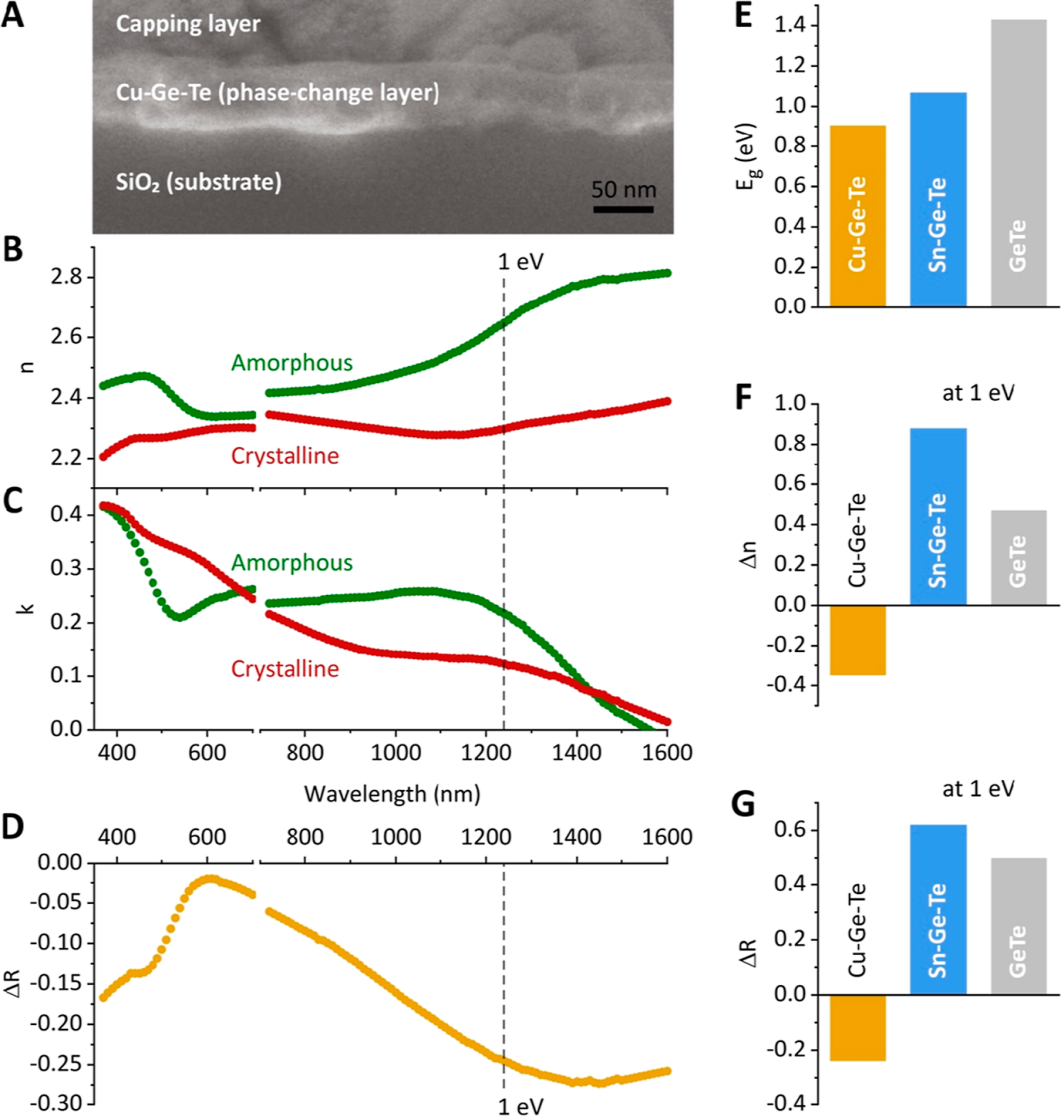
Optical characterization of CGT nanoparticle thin films. (A) Cross-sectional SEM image of CGT stack, approximately 60 nm in thickness. (B) Refractive index, (C) extinction coefficient, and (D) reflectivity contrast, spectrally resolved for amorphous and crystalline GCT thin films. (E–G) Comparison of optical properties for CGT, SGT, and GeTe thin films: band gap of amorphous thin films in (E), difference in refractive index at 1 eV in (F), and reflectivity contrast at 1 eV in (G).

The CGT nanoparticles can be deposited by spin coating on any arbitrary substrate. For this study, we choose the Si|SiO_2_ substrate and replace the nanoparticle organic ligands with GeI_2_ prior to deposition.^37^ Finally, we deposit a thin SiO_2_ capping layer to protect the spin-coated CGT film. Figure 5A shows a cross-sectional SEM image of the investigated film. We then proceed to spectroscopic ellipsometry (SE), which measures the ratio of orthogonal polarization of light reflected from the thin-film sample from which the complex refractive index with the real part, *n*, and imaginary part, *k*, can be deduced. Using the Cauchy dispersion model as the starting model, the SE data are fitted by a point-by-point algorithm to obtain precise results. We adjust the model fitting to match the film thickness within the error margin of the deposition method. The model also considers the presence of organics and a packing ratio of 0.6 (with 40% voids in the GCT nanoparticle layer), as used in our previous work on Sn–Ge–Te (SGT) quantum dot PCM.^37^Figure 5B,C plots the refractive indices and the extinction coefficients for the as-deposited CGT thin film (i.e., amorphous) and after the stack is annealed to 300 °C (i.e., crystalline). Knowing *n* and *k* for both phases, the reflectivity of amorphous and crystalline CGT (Figure S4) as well as the reflectivity contrast (Δ*R*, Figure 5D) can be calculated.^37^

We analyze the phase-change optical properties of CGT thin films, comparing Sn–Ge–Te and GeTe layers, reported previously.^32,37^ CGT exhibits a narrower band gap (Figure 5E), as estimated from the extinction coefficient of amorphous phases (Figure 5C). This appears as a benefit of CGT, providing access to longer wavelengths, including the near-IR region relevant for telecom applications.

For the amorphous CGT thin film, we observe higher refractive indexes across all measured spectra (Figure 5B). The refractive indices diverge for wavelengths above 700 nm, and at the benchmark of 1 eV, the difference in refractive index, Δ*n*, is −0.35 (Figure 5F). Negative values of Δ*n* for CGT thin films translate to a negative reflectivity contrast (Figure 5D). In general, common PCM such as GeTe and Ge_2_Sb_2_Te_5_ tend to show an increase in reflectivity upon crystallization due to density changes, which is also confirmed by the Clausius–Mossotti law.^22,52^ This also holds true for GeTe and Sn–Ge–Te quantum dot thin films, which we measured previously (Figure 5G). However, there are also a few unconventional PCMs, such as the Fe–Te and some in the Ge–Sb system, that exhibit a negative change of reflectance upon crystallization.^53,54^ Alloys that fall in the GeTe–CuTe binary line have also been found to display negative reflectivity contrast after crystallization for Cu content higher than 25 at. %.^20,22^ We see a similar trend for CGT nanoparticle thin film but for Cu doping as low as 5–7 at. % (Figure 5G). Nevertheless, thin films of GCT nanoparticles show a significant reflectivity contrast in optical properties between the amorphous and crystalline phases. The contrast becomes more pronounced for the IR region, exceeding −0.2 for the spectral range beyond 1 eV (Figure 5G). This is of particular interest in the advanced design of reflective displays, where inverted reflectivity contrast may be adopted.^55−57^ It may also be suitable for optical phase-change applications in the IR region.

## Conclusions

In this study, we explore the versatility of the amide-promoted synthesis approach in controlling the phase of ternary PCM nanoparticles by investigating the CGT system. Specifically, we present a convenient colloidal synthesis route to produce sub-10 nm CGT nanoparticles. We show accurate structure control by synthesizing CGT nanoparticles in amorphous and crystalline forms. We follow up with high-temperature structural characterization of CGT nanoparticles, showing improved amorphous phase stability and higher crystallization temperatures, which is demonstrated experimentally using in situ high-temperature XRD and is explained using local structure XAS analysis.

Furthermore, CGT thin films have been fabricated using ligand-exchanged nanoparticle inks of nanoparticles via spin coating to assess the optical properties of amorphous and crystalline thin films through an ellipsometry study. Based on this, we present an analysis, comparing the change in refractive index Δ*n*, between the amorphous and crystalline states of the nanoparticle-based thin films, extinction coefficient *k*, and band gap *E*_g_, to show the promise of CGT nanoparticles for phase-change optics. Using SE characterization, we reveal that CGT thin films have a negative reflectivity contrast as well as a pronounced change of refractive indices in the near-IR spectral region. Therefore, our work provides materials design for nanoscale PCM devices operating efficiently in the IR region, such as phase modulators, metalenses, or reflectivity IR displays. Due to the inevitable trend to scale down the dimensions of memory devices where size effects become evident,^58^ colloidal nanoparticles appear to be a convenient model system to design scaling rules of such ultrasmall PCM devices of the future.

Finally, our work offers a broader perspective, since ternary telluride colloids are of growing interest, e.g., for thermoelectric applications^59−62^ as well as for optoelectronic devices, including photodetectors and transistors.^63,64^ Due to the simplicity in depositing the colloids through spin coating or printing, ternary telluride nanoparticles may become a cost-effective alternative for those technologies. The surface of nanoparticles, although offering an additional design toolkit, needs to be better controlled in order to achieve a high packing density of thin films as well as to eliminate detrimental surface defects and to suppress oxidation processes.^65^

## Supporting Information Available

Data of experimental conditions for Cu–Ge–Te nanoparticles, X-ray diffractograms, X-ray absorption spectra with fittings, parameters, and reflectivity spectra (PDF)
