# Supplementary material for: Phase-Controlled Synthesis and Phase-Change Properties of Colloidal Cu–Ge–Te Nanoparticles
Source: Chem Mater. 2024 Jun 24;36(13):6598–607. doi: 10.1021/acs.chemmater.4c01009 (PMC11238340; doi:10.1021/acs.chemmater.4c01009)
Supplement: Supplementary file 1 — cm4c01009_si_001.pdf [file cm4c01009_si_001.pdf]

Supporting Information to

Phase Controlled Synthesis and Phase Change Properties of Colloidal Cu-Ge-Te Nanoparticles

*Dhananjeya Kumaar,[1] Matthias Can,[1] Helena Weigand,[2] Olesya Yarema,[3] Simon Wintersteller,[1] Rachel Grange,[2] Vanessa Wood,[3] and Maksym Yarema[1,]**

[1]Chemistry and Materials Design, Institute for Electronics, Department of Information Technology and Electrical Engineering, ETH Zürich, 8092 Zürich, Switzerland

[2]Optical Nanomaterial Group, Institute for Quantum Electronics, Department of Physics, ETH Zürich, 8093 Zürich, Switzerland

[3]Materials and Device Engineering, Institute for Electronics, Department of Information Technology and Electrical Engineering, ETH Zürich, 8092 Zürich, Switzerland

* yaremam@ethz.ch

Table S1. Experimental condition for the syntheses of CGT nanoparticles carried out at 260°C, and the phase analysis results from X-ray diffraction measurements.

CuI mg	GeI_2 mg	CuI mmol	GeI_2 mmol	CuI/(CuI+GeI_2)	Inj. Temp. °C	Growth time, min	Phase analysis (from XRD)
6.94	128.5	0.03644	0.39364	0.08473	260	1	amorphous
6.94	128.5	0.03644	0.39364	0.08473	260	3.5	amorphous
6.94	128.5	0.03644	0.39364	0.08473	260	5	GeTe
6.94	128.5	0.03644	0.39364	0.08473	260	10	GeTe
6.25	115.64	0.03282	0.35425	0.08478	260	20	GeTe
12.49	102.79	0.06558	0.31488	0.17237	260	1	amorphous
12.49	102.79	0.06558	0.31488	0.17237	260	3.5	amorphous
12.49	102.79	0.06558	0.31488	0.17237	260	5	amorphous
12.49	102.79	0.06558	0.31488	0.17237	260	10	GeTe
12.49	102.79	0.06558	0.31488	0.17237	260	20	GeTe
24.99	77.09	0.13122	0.23615	0.35718	260	5	amorphous
37.48	49.97	0.1968	0.15308	0.56248	260	2	amorphous
37.48	49.97	0.1968	0.15308	0.56248	260	5	amorphous
37.48	49.97	0.1968	0.15308	0.56248	260	6	amorphous
37.48	49.97	0.1968	0.15308	0.56248	260	7	amorphous
37.48	49.97	0.1968	0.15308	0.56248	260	9	amorphous
37.48	49.97	0.1968	0.15308	0.56248	260	15	amorphous
37.48	49.97	0.1968	0.15308	0.56248	260	20	Cu_2GeTe_3
37.48	49.97	0.1968	0.15308	0.56248	260	21	Cu_2GeTe_3
37.48	49.97	0.1968	0.15308	0.56248	260	25	Cu_2GeTe_3
37.48	49.97	0.1968	0.15308	0.56248	260	31	Cu_2GeTe_3
37.48	49.97	0.1968	0.15308	0.56248	260	45	Cu_2GeTe_3
37.48	49.97	0.1968	0.15308	0.56248	260	46	Cu_2GeTe_3
43.73	38.55	0.22961	0.11809	0.66037	260	5	amorphous
43.73	38.55	0.22961	0.11809	0.66037	260	10	amorphous
43.73	38.55	0.22961	0.11809	0.66037	260	20	amorphous
43.73	38.55	0.22961	0.11809	0.66037	260	45	Cu_2GeTe_3
56.22	12.85	0.2952	0.03936	0.88234	260	1	CuTe + Cu_2GeTe_3
56.22	12.85	0.2952	0.03936	0.88234	260	2	CuTe + Cu_2GeTe_3
56.22	12.85	0.2952	0.03936	0.88234	260	3	CuTe + Cu_2GeTe_3
56.22	12.85	0.2952	0.03936	0.88234	260	5	CuTe + Cu_2GeTe_3
56.22	12.85	0.2952	0.03936	0.88234	260	10	CuTe + Cu_2GeTe_3
56.22	12.85	0.2952	0.03936	0.88234	260	20	CuTe + Cu_2GeTe_3
56.22	12.85	0.2952	0.03936	0.88234	260	45	CuTe + Cu_2GeTe_3

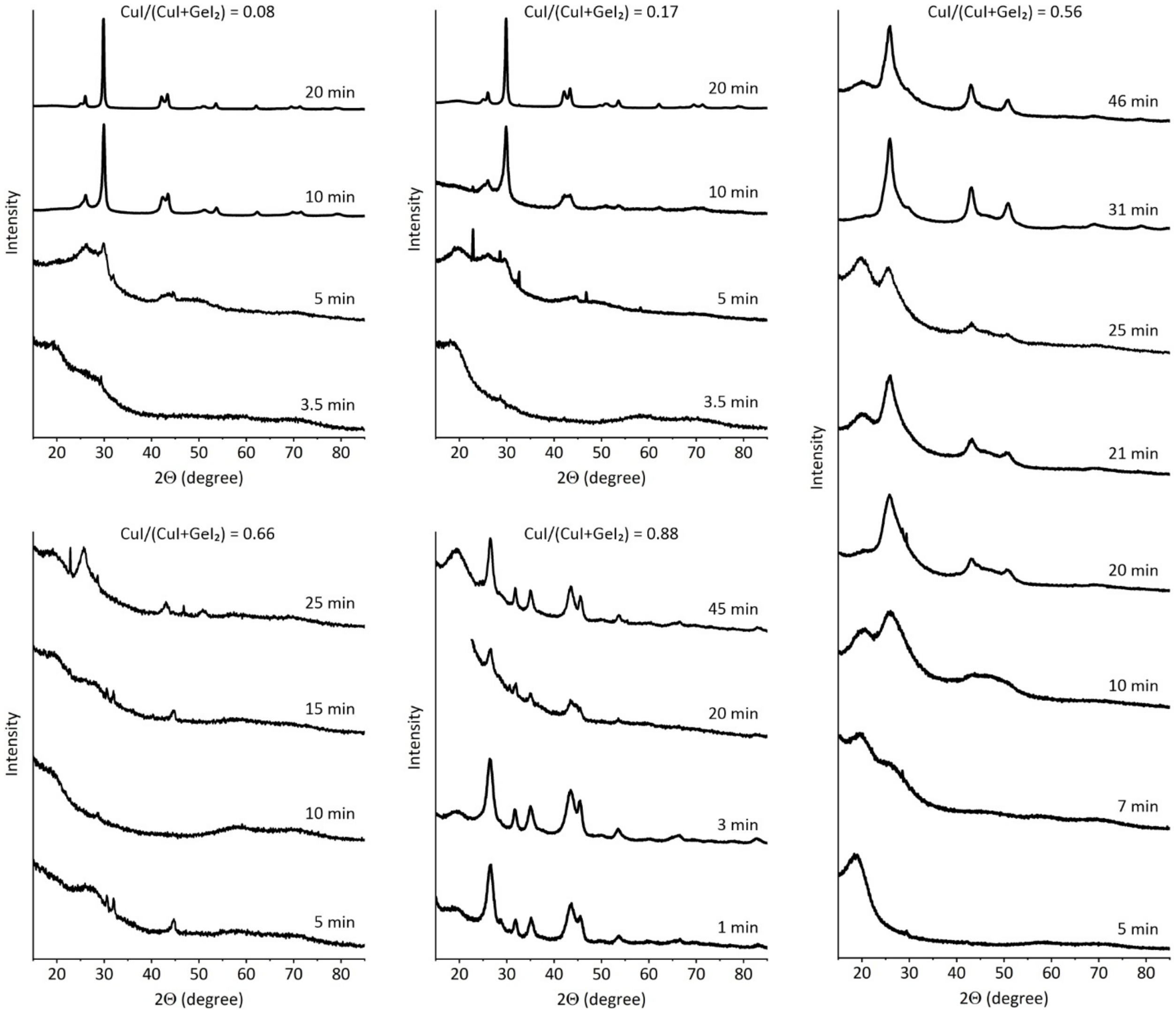

Figures S1. XRD patterns of CGT nanoparticles, synthesized during the reaction at 260°C and for various precursor ratios and reaction times, as indicated. The broad peak around 20 2Θ degree is associated with organic moiety in the sample such as ligands and remainder of solvents.

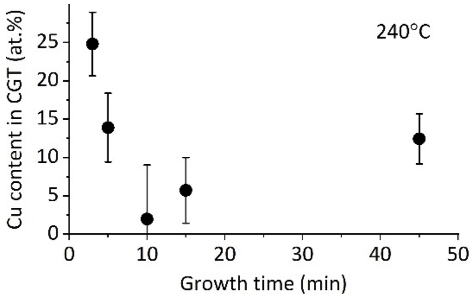

Figure S2. Cu content in CGT nanoparticles as the reaction proceeds at 240°C and for different reaction times. Initially the Cu content in the nanoparticles drops, however, at longer reaction times the Cu concentration increases because the thermodynamically favored phase is formed.

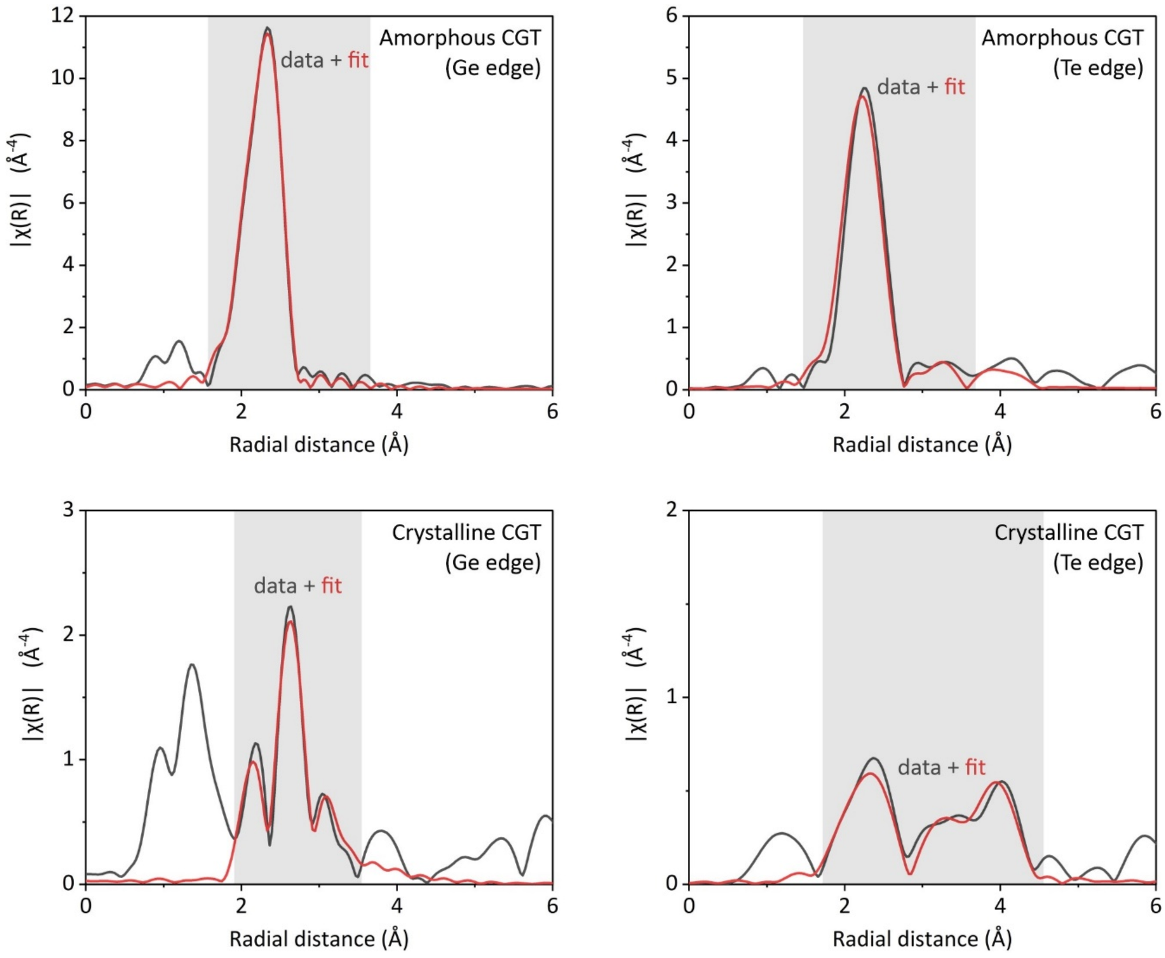

Figure S3. XAS data of amorphous and crystalline CGT nanoparticles for the Ge and Te edges. The shaded grey areas indicate the fitting windows. The composition of CGT nanoparticles is Cu:Ge:Te = 5:35:60 (at.%) and the size of CGT nanoparticles is 3.6 ± 0.6 nm.

Table S2. EXAFS fitting results of amorphous CGT nanoparticles, measured at room temperature and for the Ge and Te K-edges.

		Amorphous CGT nanoparticles							
**Edge**	**Scattering path**	**CN**		**σ^2 (10^{-3} Å^{-2})**		**Δe_0 (eV)**		**R (Å)**	
Ge edge	Ge-Ge	1.10	± 0.07	3.70	-	3.18	±0.43	2.45	±0.00
	Ge-Te1	2.14	±0.13	4.90	±0.69	3.18	±0.43	2.59	±0.00
Te edge	Cu-Te	0.61	1.51	5.67	±24.11	2.58	±18.24	2.70	±0.58
	Ge-Te1	2.14	±0.13	5.86	±24.92	2.58	±18.24	2.59	±0.00
	Te-Te1	1.00	±6.20	8.51	±36.18	2.58	±18.24	4.11	±0.33
	Te-Te2	1.00	±6.20	10.40	±44.22	2.58	±18.24	4.38	±0.54
	Te-Te Bulk	0.46	±2.13	5.67	±24.11	2.58	±18.24	3.55	±0.26
R factor		0.0148							

Table S3. EXAFS fitting results of crystalline CGT nanoparticles, measured at room temperature and for the Ge and Te K-edges.

		Crystalline CGT nanoparticles							
**Edge**	**Scattering path**	**CN**		**σ^2 (10^{-3} Å^{-2})**		**Δe_0 (eV)**		**R (Å)**	
Ge edge	Ge-Ge	0.12	± 0.07	3.70	-	5.74	±2.06	2.41	±0.03
	Ge-Te1	1.56	±0.46	9.19	±5.73	5.74	±2.06	2.83	±0.01
	Ge-Te2	1.51	±0.82	14.52	±9.05	5.74	±2.06	3.09	±0.03
Te edge	Cu-Te	0.61	1.51	10.61	±57.04	3.94	±6.93	3.00	±0.16
	Ge-Te1	1.56	±0.46	10.96	±58.93	3.94	±6.93	2.83	±0.01
	Ge-Te2	1.51	±0.82	17.33	±93.18	3.94	±6.93	3.09	±0.03
	Te-Te1	5.07	±7.08	15.91	±85.54	3.94	±6.93	4.13	±0.15
	Te-Te2	5.07	±7.08	19.45	±104.58	3.94	±6.93	4.29	±0.32
	Te-Te Bulk	0.81	±1.86	10.61	±57.05	3.94	±6.93	3.50	±0.19
R factor		0.0599							

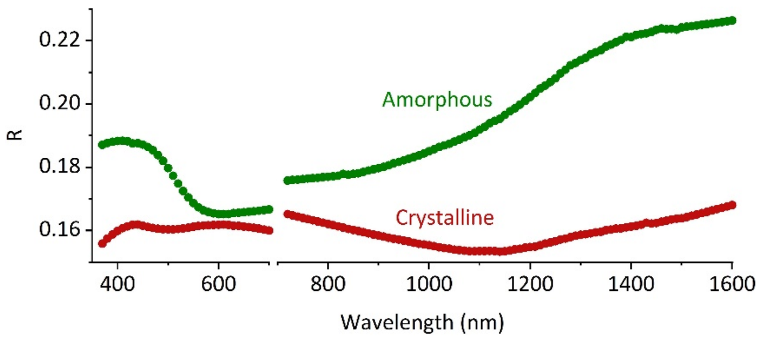

Figure S4. Reflectivity of amorphous and crystalline CGT thin films.
